# Evaluation of Prognostic Factors for Prolonged Intensive Care Unit Hospitalization After Trauma: Roles of Hemorrhagic Burden and Admission Laboratory Abnormalities

**DOI:** 10.3390/medicina62101832

**Published:** 2026-09-23

**Authors:** Yunchul Park, Do Wan Kim, Young-Goun Jo, Hyun-Seok Jang, Eui-Sung Jeong, Hyo-Sin Kim, Ji-Hyoun Kang

**Affiliations:** 1 Division of Trauma, Department of Surgery, Chonnam National University Medical School and Hospital, Gwangju 61469, Republic of Korea; 2 Department of Thoracic and Cardiovascular Surgery, Chonnam National University Medical School and Hospital, Gwangju 61469, Republic of Korea; 3 Department of Surgery, Chonnam National University Medical School and Hospital, Gwangju 61469, Republic of Korea; 4 Division of Rheumatology, Department of Internal Medicine, Chonnam National University Medical School and Hospital, Gwangju 61469, Republic of Korea

**Keywords:** trauma, prognostic factors, intensive care unit, prolonged ICU stay, massive transfusion, damage control surgery, admission laboratory abnormalities

## Abstract

*Background and Objectives*: Prolonged intensive care unit (ICU) hospitalization after trauma reflects sustained critical-care needs, but early prognostic assessment remains challenging. The primary objective was to identify factors associated with prolonged ICU hospitalization (≥7 days) using admission characteristics and treatment information available during the first 24 h. The secondary objective was to explore whether these associations differed according to a composite score of admission laboratory abnormalities (CE score). *Materials and Methods*: This retrospective cohort study included 2638 patients admitted between 1 January 2006 and 31 December 2021 from a prospectively maintained Level I trauma registry. Prolonged ICU hospitalization was defined as ICU length of stay (LOS) ≥7 days. Multivariable regression evaluated independent associations between early clinical factors and prolonged ICU hospitalization. Exploratory interaction analyses evaluated effect modification by the CE score, a composite of admission laboratory abnormalities. In-hospital deaths were retained in the primary cohort. The potential influence of in-hospital death on ICU LOS was evaluated in an additional survivor-restricted sensitivity analysis. Sensitivity analyses used ICU LOS ≥ 10 days and log-transformed ICU LOS. *Results*: Overall, 757/2638 patients (28.7%) had an ICU LOS ≥ 7 days. Older age (adjusted OR [aOR] 1.01 per year, 95% CI 1.01–1.02; *p* < 0.001), higher Injury Severity Score (aOR 1.06 per point, 95% CI 1.05–1.08; *p* < 0.001), massive transfusion (aOR 1.52, 95% CI 1.09–2.12; *p* = 0.013), and damage control surgery (aOR 2.35, 95% CI 1.60–3.46; *p* < 0.001) were independently associated with prolonged ICU hospitalization, whereas admission lactate, systolic blood pressure, and angioembolization were not. CE interactions were observed for lactate, SBP, and massive transfusion (likelihood-ratio *p* < 0.001, *p* = 0.006, and *p* = 0.037, respectively). Using the ≥10-day threshold, associations persisted for massive transfusion (aOR 1.56, 95% CI 1.10–2.22; *p* = 0.012) and DCS (aOR 1.88, 95% CI 1.27–2.79; *p* = 0.002). Among 688 hospital survivors in the additional analysis, none of the three CE interactions was statistically significant (*p* = 0.208, *p* = 0.572, and *p* = 0.574, respectively). *Conclusions*: Older age, greater injury severity, massive transfusion, and DCS were associated with prolonged ICU hospitalization after trauma. Exploratory CE interactions were not statistically significant in the survivor-restricted analysis and require further investigation. These findings do not establish the clinical utility of the CE score.

## 1. Introduction

Traumatic injury remains a leading cause of preventable death and disability worldwide, and contemporary trauma systems have increasingly improved early survival through timely hemorrhage control, hemostatic resuscitation, and coordinated critical care. As mortality decreases among patients who previously would have died from early exsanguination or refractory shock, the downstream consequences of severe injury—including prolonged organ support, infectious complications, repeated interventions, rehabilitation needs, and extended critical-care utilization—have become increasingly important determinants of the overall burden of traumatic injury. In this context, intensive care unit (ICU) length of stay (LOS) is a clinically relevant outcome for early prognostic assessment and resource planning. Identifying, early in the hospital course, patients who are likely to require prolonged ICU care could therefore help clinicians anticipate complex care needs and allocate critical-care resources more effectively. However, the factors that distinguish patients requiring relatively short ICU monitoring from those who subsequently experience prolonged ICU dependency remain incompletely characterized.

Traditionally, injury severity and early physiologic shock have been viewed as dominant determinants of critical-care requirements after trauma. Injury severity scoring systems, including the Injury Severity Score (ISS), are consistently associated with ICU admission and longer ICU courses, reflecting the cumulative anatomic burden of injury and the likelihood of requiring multi-organ support. However, anatomic injury severity alone cannot fully capture the dynamic physiologic response to traumatic injury or the degree of systemic disturbance present during early resuscitation. Markers of shock and hypoperfusion—particularly admission hypotension and elevated serum lactate—therefore offer a complementary perspective on the severity of systemic insult at presentation. Lactate is a widely used indicator of tissue hypoperfusion and metabolic stress, and higher early lactate levels have been repeatedly associated with worse outcomes in critically ill and injured populations, including greater need for intensive resuscitation and critical-care support [1,2].

Beyond physiologic shock, hemorrhagic burden and treatment intensity represent additional dimensions of severe trauma that may influence subsequent ICU trajectories. Hemorrhage remains a leading cause of potentially preventable death after injury, and modern damage control resuscitation emphasizes balanced transfusion, rapid hemorrhage control, and minimization of crystalloid exposure [3,4,5]. Massive transfusion, commonly defined as ≥10 units of packed red blood cells within 24 h [6], reflects severe hemorrhage and physiologic stress and has been associated with increased organ dysfunction among trauma survivors [7]. Similarly, damage control surgery (DCS) is performed in profoundly unstable patients to achieve rapid control of bleeding and contamination but may be followed by a prolonged critical-care course involving staged operations, continued resuscitation, and open abdomen management [8,9]. Angioembolization has likewise become an important hemorrhage-control strategy for selected injuries and may identify patients with substantial hemorrhagic burden requiring complex multidisciplinary management [10,11].

Despite these well-recognized determinants, an important prognostic challenge remains: patients with similar injury severity, early physiologic derangement, and resuscitation requirements may nevertheless experience markedly different ICU trajectories. This clinical heterogeneity suggests that prolonged critical-care utilization may be influenced not only by anatomic injury and the magnitude of acute physiologic shock but also by patient-related vulnerability.

Frailty provides one conceptual framework for understanding such vulnerability, although formal frailty assessment is often difficult during emergency trauma evaluation because information regarding pre-injury function, mobility, cognition, and independence may not be immediately available. Routine laboratory measures have therefore been investigated as pragmatic surrogate markers when formal frailty assessments are unavailable [12,13]. These measures cannot substitute for validated multidimensional frailty instruments, but they may capture aspects of underlying physiologic vulnerability that are readily identifiable at the time of admission. Consistent with this concept, pre-injury condition has been identified as an important factor associated with prolonged ICU stay in large trauma registry studies [14].

Among candidate laboratory indicators, hypoalbuminemia, anemia, and renal dysfunction are routinely assessable during the initial evaluation and have been investigated in relation to adverse outcomes in trauma and critical illness [15,16,17,18,19,20]. Hypoalbuminemia has been linked to increased morbidity, prolonged ICU and hospital stay, and poorer outcomes after trauma and critical illness [15,16,17]. Likewise, admission anemia has been associated with adverse outcomes in trauma patients [18,19]. Acute kidney injury has also been associated with increased mortality and longer hospital stays in critically ill trauma patients [20]. These findings support considering hematologic and renal abnormalities in the assessment of trauma patients, although admission measurements cannot reliably distinguish pre-existing vulnerability from acute injury-related changes. Importantly, none of these abnormalities is specific to frailty or chronic vulnerability, particularly in the setting of acute traumatic injury.

However, although individual laboratory markers such as albumin and hemoglobin have been studied in various clinical settings, their contribution to prognostic heterogeneity in ICU trajectories after trauma remains unclear, particularly with respect to effect modification. Most studies evaluating prolonged ICU stay have focused primarily on injury pattern, intubation status, physiologic indices, or other individual risk factors and have generally examined these variables as independent predictors [14].

To address this gap, our primary objective was to identify factors associated with prolonged ICU hospitalization, defined as ICU LOS ≥ 7 days. As a secondary exploratory objective, we examined whether a composite score based on admission hypoalbuminemia, anemia, and reduced renal function modified the associations of admission lactate, systolic blood pressure, and massive transfusion with prolonged ICU hospitalization. Sensitivity analyses using a more stringent ICU LOS threshold and continuous ICU LOS modeling were performed to assess the robustness of the primary associations.

## 2. Materials and Methods

We conducted a retrospective cohort study using data from a prospectively maintained trauma registry at a regional Level I trauma center, including patients admitted between 1 January 2006 and 31 December 2021. The registry systematically collects demographic characteristics, injury-related variables, initial physiologic parameters, laboratory findings, therapeutic interventions, and clinical outcomes for patients admitted after traumatic injury. The present study was designed to evaluate factors available during the early phase of trauma care that were associated with subsequent prolonged ICU hospitalization. The study protocol was reviewed and approved by the institutional review board. The requirement for informed consent was waived because of the retrospective nature of the study and the use of routinely collected clinical data.

All patients admitted for traumatic injury and registered in the institutional trauma registry between 1 January 2006 and 31 December 2021 were screened for eligibility. Patients with available ICU length-of-stay (LOS) data were included in the primary analytic cohort. Of 4106 trauma registry records screened, 1468 patients without available ICU LOS data were excluded, resulting in a final analytic cohort of 2638 patients. The patient selection process is summarized in Supplementary Figure S1. Transfers and readmissions were not excluded. Missing laboratory values were not considered exclusion criteria for the overall study cohort; patients with missing covariate data were excluded only from analyses requiring those variables through complete-case analysis.

All eligible patients meeting the study criteria were included in the analytic cohort. For multivariable analyses, complete-case analysis was performed according to the availability of covariates included in each model; therefore, the number of patients contributing to individual models could vary according to covariate completeness. Variable-specific data availability and missingness for the primary cohort and the additional mortality sensitivity cohort are reported separately in Supplementary Table S1.

## 3. Outcome Definition

The primary outcome was prolonged ICU hospitalization, defined a priori as an ICU length of stay (ICU LOS) of ≥7 days. This threshold was selected as a clinically meaningful marker of sustained critical care utilization and was additionally evaluated using a ≥10-day threshold and continuous ICU LOS in sensitivity analyses. ICU LOS was obtained from the institutional registry and represented the duration of the first ICU admission during the index hospitalization. Subsequent ICU readmissions were not included in the calculation of ICU LOS. This binary outcome was selected to identify patients requiring sustained critical care beyond the early post-injury period and to provide a clinically interpretable measure of prolonged ICU resource utilization.

Because any single cutoff for prolonged ICU stay may be influenced by institutional practice patterns and patient case mix, two complementary sensitivity analyses were performed to evaluate the robustness of the primary findings. First, prolonged ICU hospitalization was redefined using a more stringent threshold of ≥10 ICU days. Second, ICU LOS was analyzed as a continuous outcome after logarithmic transformation to account for its expected right-skewed distribution. The use of both an alternative binary threshold and a continuous outcome was intended to determine whether the observed associations were dependent on the specific 7-day definition used for the primary analysis.

## 4. Exposure Variables and Covariates

Candidate exposure variables and covariates were selected to represent distinct dimensions of early post-trauma risk that could be assessed during the initial phase of care. These included baseline demographic and injury characteristics, early physiologic derangement, and markers of hemorrhagic burden and treatment intensity. Age and Injury Severity Score (ISS) were included to account for baseline demographic risk and overall anatomic injury severity, respectively.

Early physiologic derangement was characterized using systolic blood pressure (SBP) and serum lactate measured on admission. These variables were selected as routinely available indicators of hemodynamic compromise and global tissue hypoperfusion during the initial assessment of injured patients. Both variables were evaluated using values obtained on arrival, thereby focusing the analysis on information available early in the clinical course rather than on subsequent physiologic changes occurring during ICU treatment.

Hemorrhagic burden and the intensity of early hemorrhage-control treatment were represented by massive transfusion, damage control surgery (DCS), and angioembolization. Massive transfusion was defined as transfusion of ≥10 units of packed red blood cells within the first 24 h after admission. DCS and angioembolization were coded as binary variables indicating whether each intervention was performed within the same 24 h period; procedures performed after this period were not included in these exposure definitions. Thus, the analysis combined admission measurements with treatment information collected during the initial 24 h. The outcome was a first ICU stay lasting ≥7 days, rather than an additional seven days after the 24 h exposure-ascertainment window. Together, these variables were intended to capture complementary aspects of early injury severity, physiologic stress, and treatment burden potentially associated with subsequent ICU utilization. Accordingly, the analyses should be interpreted as an early prognostic assessment based on information available during the first 24 h after admission, rather than as a prediction model applicable at the time of admission.

## 5. Composite Admission Laboratory Abnormalities Score

Admission laboratory abnormalities were summarized using the composite vulnerability score (CE score) derived from three routinely available laboratory measurements obtained at admission. The score was developed for the present analysis as a pragmatic summary of three laboratory abnormalities present at admission. It was not intended to represent a validated frailty instrument, to establish the presence of pre-existing frailty, or to directly quantify pre-injury functional reserve. Rather, the CE score was used to provide a simple laboratory-based context in which the prognostic significance of early post-trauma physiologic and treatment-related factors could be evaluated.

The CE score ranged from 0 to 3, with one point assigned for each of the following abnormalities present on admission:

1. Hypoalbuminemia, defined as serum albumin <3.5 g/dL;
2. Anemia, defined as hemoglobin ≤11 g/dL; and
3. Reduced renal function, defined as an estimated glomerular filtration rate (eGFR) <60 mL/min/1.73 m^2^, calculated using the CKD-EPI equation.

These components and their prespecified thresholds were selected based on the clinical literature discussed above [15,16,17,18,19,20], clinical interpretability, and their routine availability during the initial evaluation of trauma patients. One point was assigned to each abnormality to provide a simple, transparent count of the specified admission laboratory abnormalities. Equal weighting was not intended to imply equivalent biological importance or prognostic contributions, and the component thresholds were not intended to define pre-existing frailty. Because admission albumin, hemoglobin, and eGFR may be influenced by acute hemorrhage, fluid resuscitation, shock, or acute kidney injury, the score may reflect both pre-existing conditions and acute injury-related changes. Accordingly, the CE score was used as an exploratory summary of the admission laboratory profile rather than a direct measure of pre-injury physiologic reserve.

Importantly, the CE score was not evaluated as a stand-alone clinical prediction model. Instead, it was incorporated primarily as a potential effect modifier to examine whether the prognostic associations of early physiologic shock and hemorrhage-related treatment intensity with prolonged ICU hospitalization differed according to the patient’s admission laboratory profile. Interaction terms between the CE score and admission lactate, systolic blood pressure, and massive transfusion were therefore evaluated in the regression analyses. Effect estimates at CE scores of 0, 1, 2, and 3 were subsequently derived from the interaction models to characterize the direction and magnitude of the observed effect modification.

Accordingly, the purpose of the CE score was not to replace established measures of injury severity or physiologic derangement but to determine whether the prognostic information conveyed by these conventional early trauma indicators varied across CE-score levels. The CE score should therefore be regarded as an exploratory, internally derived composite rather than a validated clinical instrument.

## 6. Statistical Analysis

All statistical analyses were performed using Stata version 19 (StataCorp LLC, College Station, TX, USA). Continuous variables were summarized as means with standard deviations or medians with interquartile ranges, as appropriate, and compared using Student’s *t*-test or the Mann–Whitney U test. Categorical variables were compared using the chi-square or Fisher’s exact test. Multivariable logistic regression models were constructed to evaluate independent associations with prolonged ICU hospitalization. Covariates included age, ISS, admission physiologic parameters (SBP and serum lactate), and markers of hemorrhagic burden and treatment intensity (massive transfusion, DCS, and angioembolization). Sensitivity analyses included logistic regression using an alternative ICU LOS threshold (≥10 days) and multivariable linear regression using log-transformed ICU LOS as a continuous outcome. To explore the potential competing effect of in-hospital death on ICU LOS, an additional mortality sensitivity analysis was performed using records for which mortality status and timing could be evaluated. In-hospital mortality was examined according to ICU LOS category, and the multivariable and interaction analyses were repeated after restriction to patients who survived to hospital discharge. All statistical tests were two-sided, and *p* values < 0.05 were considered statistically significant.

## 7. Effect Modification Analysis

Effect modification analyses were performed to determine whether the prognostic associations of early physiologic shock and hemorrhage-related treatment intensity with prolonged ICU hospitalization varied according to the degree of admission laboratory-based abnormalities. Separate interaction terms were evaluated between the CE score and three prespecified exposures: admission lactate, systolic blood pressure (SBP), and massive transfusion. These exposures were selected to represent complementary aspects of early post-trauma physiologic derangement and hemorrhagic burden.

For the interaction analyses, CE was treated as a numeric variable ranging from 0 to 3 to provide a parsimonious, one-degree-of-freedom assessment of an ordered trend in effect modification. This specification assumes a constant change in the exposure-specific log odds ratio for each one-point increase in CE. CE-specific estimates derived from these models are therefore conditional on this linear interaction assumption. CE-specific adjusted odds ratios and corresponding 95% confidence intervals were calculated from the fitted interaction models at CE scores of 0, 1, 2, and 3.

Evidence of effect modification was assessed using likelihood ratio tests comparing nested multivariable models with and without the corresponding interaction term. A statistically significant likelihood ratio test was interpreted as evidence that the association between the exposure and prolonged ICU hospitalization differed according to the CE score rather than remaining constant across vulnerability levels.

For interactions meeting the prespecified threshold for statistical significance, stratum-specific effect estimates and corresponding 95% confidence intervals were calculated at CE scores of 0, 1, 2, and 3. These estimates were used to characterize the direction and magnitude of the interaction and to facilitate clinical interpretation of prognostic heterogeneity across increasing levels of laboratory-based abnormalities. Interaction patterns were additionally presented graphically to illustrate changes in the estimated associations across CE-score levels.

## 8. Results

### 8.1. Patient Characteristics According to Prolonged ICU Stay

Among 2638 trauma patients with available ICU length-of-stay data who met the study eligibility criteria, 28.7% experienced prolonged ICU hospitalization, defined a priori as an ICU LOS of ≥7 days. Patients were stratified according to this predefined threshold, and their baseline demographic, injury-related, physiologic, and treatment characteristics are summarized in Table 1. Of the 2638 patients in the primary cohort, 2146 (81.3%) had complete data for all seven covariates included in the primary multivariable model: age, ISS, admission lactate, admission SBP, massive transfusion, DCS, and angioembolization. The remaining 492 patients (18.7%) were excluded from this model because of missing covariate data. Among the complete cases, 611 (28.5%) had ICU LOS ≥ 7 days. The same 2146 patients contributed to the ≥10-day sensitivity model, with 427 (19.9%) meeting this outcome.

Patients with prolonged ICU hospitalization generally presented with a greater overall burden of traumatic injury and early physiologic derangement than those with ICU stays of <7 days. In particular, the prolonged ICU group had higher Injury Severity Scores (ISS), indicating greater anatomic injury severity. Admission lactate concentrations were also higher, whereas systolic blood pressure (SBP) on arrival was lower among patients who subsequently required prolonged ICU care.

Markers of hemorrhagic burden and treatment intensity also differed between the two groups. Massive transfusion, damage control surgery (DCS), and angioembolization were more frequently observed among patients with prolonged ICU hospitalization. Thus, patients requiring an ICU stay of ≥7 days were characterized not only by greater injury severity and physiologic derangement at presentation but also by more frequent use of major hemorrhage-control interventions during the early phase of trauma care. As expected from the outcome definition, the observed duration of ICU hospitalization was substantially longer in the prolonged ICU group than in patients with ICU LOS < 7 days.

### 8.2. Distribution of ICU Stay Duration

The overall distribution of ICU LOS is shown in Figure 1. ICU LOS demonstrated a markedly right-skewed distribution, with most patients requiring relatively short periods of critical care and a progressively smaller proportion remaining in the ICU for extended periods. A distinct subgroup nevertheless experienced substantially prolonged ICU hospitalization.

Given this non-normal distribution, ICU LOS was evaluated primarily as a dichotomous clinical outcome using the prespecified threshold of ≥7 days. The skewed distribution also provided the rationale for complementary analyses using both a more stringent binary threshold and a log-transformed continuous measure of ICU LOS. These alternative approaches were subsequently used to assess whether the observed prognostic associations were dependent on the primary 7-day cutoff.

### 8.3. Early Prognostic Associations with Prolonged ICU Stay (≥7 Days)

Multivariable logistic regression was performed to determine which early demographic, injury-related, physiologic, and treatment-related factors remained independently associated with prolonged ICU hospitalization after simultaneous adjustment for the other variables included in the model (Table 2). Adjusted effect estimates and their corresponding 95% confidence intervals are also displayed in Figure 2.

Older age, greater anatomic injury severity, massive transfusion, and DCS were independently associated with an increased likelihood of ICU hospitalization lasting ≥7 days. Among the variables included in the model, DCS demonstrated the largest adjusted relative association with the primary outcome (adjusted OR 2.35, 95% CI 1.60–3.46; *p* < 0.001). Patients undergoing DCS therefore had more than twice the adjusted odds of prolonged ICU hospitalization compared with those who did not undergo DCS. Massive transfusion was also independently associated with prolonged ICU stay (adjusted OR 1.52, 95% CI 1.09–2.12; *p* = 0.013).

Both age and ISS showed statistically significant associations when modeled as continuous variables. Each additional year of age was associated with increased odds of prolonged ICU hospitalization (adjusted OR 1.01 per year, 95% CI 1.01–1.02; *p* < 0.001), while each additional ISS point was associated with a 6% increase in the adjusted odds of ICU LOS ≥ 7 days (adjusted OR 1.06 per point, 95% CI 1.05–1.08; *p* < 0.001). The association with ISS was therefore maintained across the range of injury severity rather than being restricted to a specific injury-severity category.

In contrast, several variables that differed between the prolonged and shorter ICU-stay groups did not retain independent associations after multivariable adjustment. Admission lactate was not independently associated with prolonged ICU hospitalization after accounting for age, ISS, and the other clinical and treatment-related covariates. Similarly, admission SBP and angioembolization were not independently associated with the primary outcome in the adjusted model. Thus, although physiologic shock markers and hemorrhage-control interventions differed between groups at the descriptive level, their adjusted prognostic associations were not uniform.

### 8.4. Sensitivity Analysis Using Alternative Prolonged ICU Threshold (≥10 Days)

To evaluate whether the primary findings depended on the prespecified 7-day definition of prolonged ICU hospitalization, the multivariable analysis was repeated using a more stringent threshold of ICU LOS ≥ 10 days (Table 3). The overall pattern of associations was consistent with that observed in the primary analysis.

Older age and higher ISS remained independently associated with prolonged ICU hospitalization using the ≥10-day threshold. Massive transfusion and DCS also retained independent associations with this more stringent definition of prolonged ICU stay. Conversely, admission lactate, admission SBP, and angioembolization remained non-significant after multivariable adjustment. The consistency of these findings across the 7-day and 10-day thresholds indicated that the principal associations were not restricted to the specific cutoff selected for the primary outcome.

ICU LOS was additionally evaluated as a continuous outcome to avoid reliance on any single dichotomous threshold. Because the distribution of ICU LOS was markedly right-skewed, LOS was log-transformed before analysis. In the continuous-outcome sensitivity analysis (n = 1423), higher ISS was associated with a 2.13% increase in ICU LOS per point (95% CI, 1.64–2.61%; *p* < 0.001), while massive transfusion and DCS were associated with 15.76% (95% CI, 2.56–30.67%; *p* = 0.018) and 32.51% (95% CI, 13.99–54.04%; *p* < 0.001) longer ICU LOS, respectively. Admission lactate was associated with a 1.77% decrease in ICU LOS per mmol/L (95% CI, −3.02 to −0.51%; *p* = 0.006). Age, admission SBP, and angioembolization were not significantly associated with continuous ICU LOS (*p* = 0.089, *p* = 0.393, and *p* = 0.781, respectively) (Figure 3).

The additional mortality sensitivity cohort included 881 patients, of whom 570 had ICU LOS < 7 days and 311 had ICU LOS ≥ 7 days. In-hospital mortality in these groups was 43/570 (7.5%) and 49/311 (15.8%), respectively. Of the 881 patients, 769 had complete data for all covariates required for the additional analyses, whereas 112 were excluded because of unavailable covariate data (Supplementary Table S1). Among the 769 complete cases, 81 died during hospitalization, leaving 688 hospital survivors for the survivor-restricted analysis; 235/688 (34.2%) had ICU LOS ≥ 7 days. In the survivor-restricted multivariable analysis, older age, higher ISS, admission lactate, massive transfusion, DCS, and angioembolization were associated with prolonged ICU stay, whereas admission SBP was not (Supplementary Table S2). The CE interactions with lactate, SBP, and massive transfusion were not statistically significant among hospital survivors (*p* = 0.208, *p* = 0.572, and *p* = 0.574, respectively).

### 8.5. Prognostic Heterogeneity by Laboratory-Based Abnormalities

We next examined whether the associations between early physiologic or hemorrhage-related factors and prolonged ICU hospitalization varied according to baseline laboratory-based abnormalities. Interaction analyses were performed using the composite CE score to evaluate potential effect modification of the associations of admission lactate, SBP, and massive transfusion with ICU LOS ≥ 7 days. Among patients with available CE-score and ICU LOS data, 25 (2.6%) had CE = 0, 649 (66.4%) had CE = 1, 277 (28.4%) had CE = 2, and 26 (2.7%) had CE = 3. Prolonged ICU hospitalization occurred in 7/25 (28.0%), 198/649 (30.5%), 107/277 (38.6%), and 11/26 (42.3%) patients, respectively.

Likelihood ratio testing demonstrated statistically significant interactions between the CE score and each of these early clinical factors (Table 4). The interaction between the CE score and admission lactate was particularly strong (LR χ^2^ = 44.13, *p* < 0.001), providing evidence that the association between admission lactate and prolonged ICU hospitalization was not constant across levels of baseline laboratory-based abnormalities. Significant interaction was also observed between the CE score and admission SBP and between the CE score and massive transfusion, indicating heterogeneity in their relative associations with prolonged ICU hospitalization according to the CE score.

To further characterize these interactions, associations were examined after stratification by CE score (Table 5). For admission lactate, the association between increasing lactate concentration and prolonged ICU hospitalization was greatest among patients with CE = 0. The magnitude of this association progressively decreased across higher CE-score categories. Consequently, the relative association of elevated admission lactate with prolonged ICU hospitalization was attenuated among patients with greater baseline laboratory-based abnormalities.

A similar pattern was observed for admission SBP. Lower SBP was more strongly associated with prolonged ICU hospitalization at lower CE-score levels, whereas the magnitude of this association became progressively less pronounced as the CE score increased. Thus, the prognostic association of an early hemodynamic measure also differed according to the patient’s baseline abnormality profile.

The association between massive transfusion and prolonged ICU hospitalization likewise varied across CE-score strata. Massive transfusion showed larger relative effect estimates at lower CE levels, with progressively smaller relative estimates at higher levels of laboratory-based abnormalities. The direction of this pattern was therefore consistent with those observed for lactate and SBP, despite these variables representing different aspects of the early post-trauma clinical state.

The interaction patterns are graphically presented in Figure 4 and Figure 5. Across the examined physiologic and hemorrhage-related factors, the relative associations with prolonged ICU hospitalization were generally greatest among patients with low laboratory-based abnormalities and attenuated as the CE score increased.

## 9. Discussion

In this retrospective cohort study, we characterized the early prognostic profile associated with prolonged ICU hospitalization after trauma. Older age, greater injury severity, massive transfusion, and damage control surgery (DCS) were independently associated with prolonged ICU hospitalization, whereas admission lactate and systolic blood pressure did not retain independent associations after multivariable adjustment. Importantly, the prognostic associations of selected physiologic and hemorrhage-related factors varied according to laboratory-based abnormalities. Together, these findings suggest that prolonged ICU utilization after trauma reflects the combined influence of host characteristics, anatomic injury burden, and treatment intensity rather than any single physiologic measurement at presentation.

Physiologic shock at presentation has long been recognized as an important determinant of adverse outcomes after trauma, particularly mortality and organ failure [21,22,23]. In the present study, patients with prolonged ICU hospitalization had higher admission lactate levels and lower systolic blood pressure in unadjusted comparisons. However, neither variable retained an independent association with prolonged ICU stay after adjustment for age, injury severity, massive transfusion, DCS, and angioembolization. This attenuation suggests that isolated admission physiology may provide limited additional prognostic information for prolonged ICU utilization once the broader injury and treatment context is considered. Nevertheless, the absence of an independent main effect does not imply that these variables are clinically unimportant. Lactate and systolic blood pressure remain central to early resuscitation assessment, and our interaction analyses indicate that their prognostic associations with prolonged ICU stay may differ according to laboratory-based abnormalities. The main-effect and interaction findings should therefore be interpreted as complementary rather than contradictory.

Hemorrhage remains a central determinant of early morbidity after trauma, and the need for massive transfusion or DCS reflects severe bleeding, physiologic instability, and the requirement for aggressive life-saving intervention [24,25,26,27]. In our cohort, both massive transfusion and DCS were independently associated with prolonged ICU hospitalization, whereas angioembolization was not independently associated after multivariable adjustment. These findings should not be interpreted as evidence that massive transfusion or DCS directly causes prolonged ICU stay. Rather, these interventions likely function as markers of severe hemorrhagic burden, complex injury patterns, and treatment intensity. DCS may additionally be followed by staged operations, open-abdomen management, and continued resuscitation, potentially contributing to extended critical-care requirements. From a prognostic perspective, treatment-related variables may therefore capture dimensions of acute injury severity and clinical complexity that are not fully represented by admission physiology alone.

A central finding of this study was the prognostic heterogeneity observed across the CE score strata. The associations of admission lactate, systolic blood pressure, and massive transfusion with prolonged ICU hospitalization differed according to the composite admission laboratory-based abnormalities score. Contrary to our initial hypothesis, the relative associations of these variables were progressively attenuated at higher CE levels. For example, the association between increasing lactate and prolonged ICU stay was strongest among patients with CE = 0 and became weaker across higher CE categories, with similar patterns observed for lower systolic blood pressure and massive transfusion.

Several factors could potentially contribute to this pattern. Patients with higher CE scores may have a higher underlying risk of prolonged ICU hospitalization irrespective of the magnitude of individual acute physiologic abnormalities, thereby reducing the relative discrimination provided by additional shock- or hemorrhage-related factors. However, this interpretation remains speculative because absolute baseline risks across vulnerability strata were not the primary focus of the interaction analysis. Statistical considerations may also contribute, including smaller sample sizes and event counts at higher CE levels and consequently less precise stratum-specific estimates. In addition, because the CE score was derived from laboratory measurements obtained at admission, its components may have been influenced by acute hemorrhage, hemodilution, shock, or kidney injury rather than exclusively reflecting pre-injury physiologic reserve.

Accordingly, the interaction findings should be interpreted as evidence of statistical effect modification and prognostic heterogeneity rather than proof of a specific biological interaction. The CE score should be regarded as an exploratory summary of admission laboratory abnormalities rather than a validated measure of pre-existing frailty or a clinical prediction instrument. Predictive performance and clinical utility were not assessed in this study, and the findings do not support using the CE score for individual risk prediction or clinical decision-making. Because CE was modeled numerically, the progressive pattern in the CE-specific estimates partly reflects the imposed linear interaction structure and should not be regarded as independent evidence of a monotonic biological gradient. Admission laboratory abnormalities may reflect both pre-existing conditions and acute changes related to trauma and resuscitation. In particular, a single reduced admission eGFR does not establish chronic kidney disease and may reflect acute kidney injury [28]. Established frailty phenotypes incorporate clinical and functional characteristics, such as unintentional weight loss, weakness, and slow walking speed, that are not captured by the CE score [29]. Accordingly, the CE score summarizes the burden of admission laboratory abnormalities without distinguishing chronic vulnerability from acute injury-related changes. Persistent inflammatory and immunosuppressive states after severe injury provide a biologically plausible context for heterogeneous recovery trajectories, but these mechanisms were not directly evaluated in the present study [30]. Future studies incorporating validated pre-injury frailty measures, pre-injury or longitudinal laboratory data, direct inflammatory biomarkers, and external cohorts are needed to investigate the biological basis and reproducibility of the observed interactions.

From a clinical perspective, our findings emphasize that prognostic assessment of prolonged ICU utilization should incorporate multiple dimensions of early trauma severity. Age and ISS provide information regarding host characteristics and anatomic injury burden, whereas massive transfusion and DCS may identify patients with particularly severe hemorrhage and complex treatment requirements. Admission lactate and systolic blood pressure remain essential measures for immediate resuscitation, but their independent prognostic contribution to subsequent prolonged ICU stay was limited after adjustment for these broader clinical factors. The observed interactions further suggest that the prognostic information conveyed by individual acute markers may not be uniform across patients with different laboratory-based abnormality profiles. At present, however, these findings should not be used to guide individual treatment decisions or ICU triage because the CE score has not been externally validated and this study was not designed to develop a clinical prediction model. Rather, the findings provide a basis for future studies evaluating integrated approaches to early prognostic stratification after trauma.

Beyond routinely available clinical and laboratory variables, emerging biomarker-based approaches may provide additional information for early risk stratification. Studies in sepsis have suggested that combining established clinical scores with inflammatory indices may improve prognostic assessment, while circulating microRNAs and plasma gelsolin have also emerged as potential prognostic biomarkers. However, whether integration of such biomarkers with routine clinical measures improves the prediction of prolonged ICU hospitalization after trauma remains unknown and requires prospective validation [31,32].

Several limitations should be considered when interpreting these findings. First, this was a retrospective analysis of a prospectively maintained registry from a single Level I trauma center, and institutional differences in ICU admission, discharge, hemorrhage-control strategies, and critical-care practices may limit generalizability. Given the retrospective observational design, the observed associations should not be interpreted as causal relationships. Second, ICU LOS is influenced not only by injury severity and physiologic recovery but also by complications, treatment decisions, bed availability, and discharge practices. Third, treatment-related exposures were assessed during the first 24 h after admission and may be subject to confounding by indication and time-related bias. Because patients who died within this period were not excluded from the primary cohort, the opportunity to receive treatment or meet the massive transfusion threshold may have differed according to survival time. Treatment decisions also depended on evolving clinical status. These temporal relationships were not explicitly modeled, and the observed treatment-related associations should therefore be interpreted cautiously as prognostic rather than causal. Death may shorten observed ICU LOS; therefore, a stay of <7 days should not necessarily be interpreted as a favorable outcome. In the survivor-restricted sensitivity analysis, the CE interactions were not statistically significant, warranting cautious interpretation of the primary interaction findings. Restriction to hospital survivors conditions on survival to discharge and does not fully resolve the problem of competing events. Fourth, complete-case analysis may have introduced selection bias if patients with complete observations differed systematically from those with missing data. Finally, the CE score was constructed from admission laboratory measurements and has not been externally validated. Its components may reflect both pre-existing conditions and acute injury-related changes, as admission albumin, hemoglobin, and renal function may be affected by acute hemorrhage, fluid resuscitation, transfusion, shock, and acute kidney injury. Therefore, the CE score should be interpreted as an exploratory summary of admission laboratory abnormalities rather than a measure of pre-injury frailty or physiological reserve. In addition, the relatively small numbers of patients and outcome events in the higher CE-score categories may have resulted in imprecise estimates and limited power for interaction analyses. The observed interactions should therefore be considered exploratory and require validation in independent trauma cohorts before the CE score can be considered for individual risk prediction or clinical decision-making.

## 10. Conclusions

In conclusion, prolonged ICU hospitalization after trauma was independently associated with older age, greater injury severity, massive transfusion, and DCS. Exploratory analyses suggested possible heterogeneity in selected prognostic associations across CE levels; however, these interactions were not statistically significant in the survivor-restricted analysis. Accordingly, the CE-related findings should be considered exploratory and require external validation before any clinical application of the CE score.

## Figures and Tables

**Figure 1:**
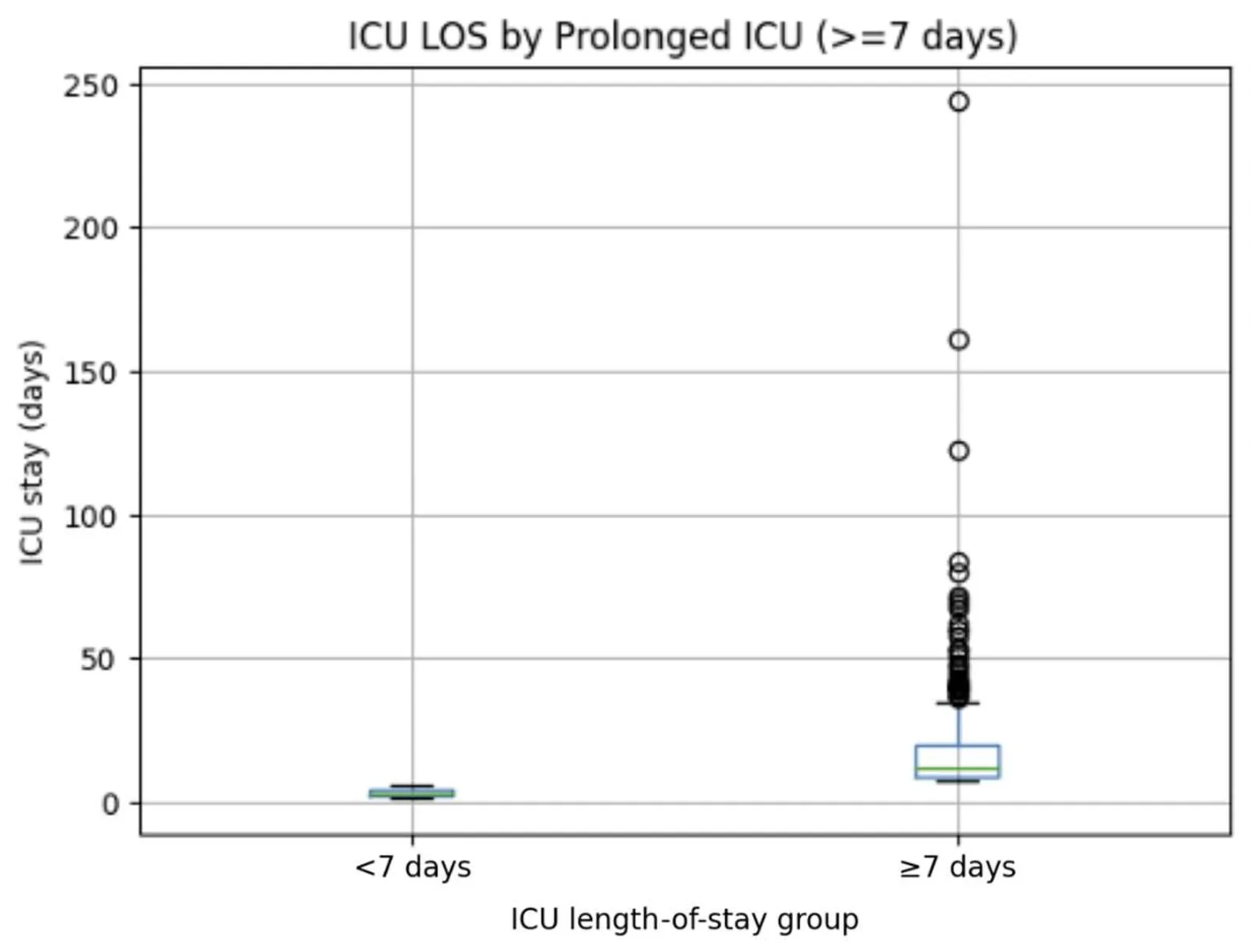
Distribution of ICU length of stay according to prolonged ICU hospitalization status (≥7 days). Boxplots show ICU length of stay among patients with stays of <7 days and ≥7 days. Individual points beyond the whiskers represent outliers. Abbreviations: ICU, intensive care unit; LOS, length of stay.

**Figure 2:**
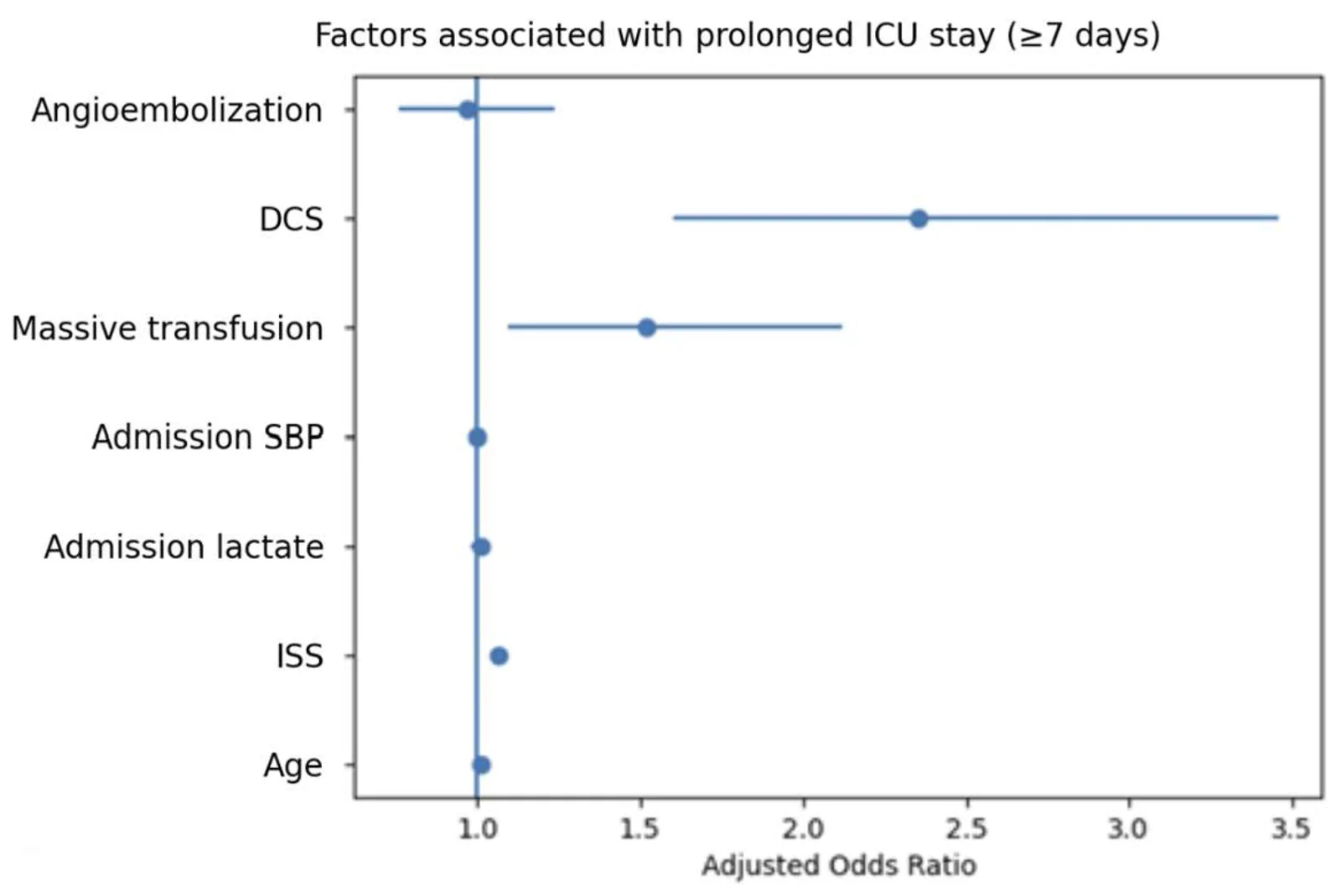
Early factors associated with prolonged ICU hospitalization (≥7 days). Points represent adjusted odds ratios, and horizontal lines indicate 95% confidence intervals from the multivariable logistic regression model. The vertical reference line indicates an odds ratio of 1. Estimates were mutually adjusted for age, ISS, admission lactate, admission SBP, massive transfusion, DCS, and angioembolization. Abbreviations: DCS, damage-control surgery; ICU, intensive care unit; ISS, Injury Severity Score; SBP, systolic blood pressure.

**Figure 3:**
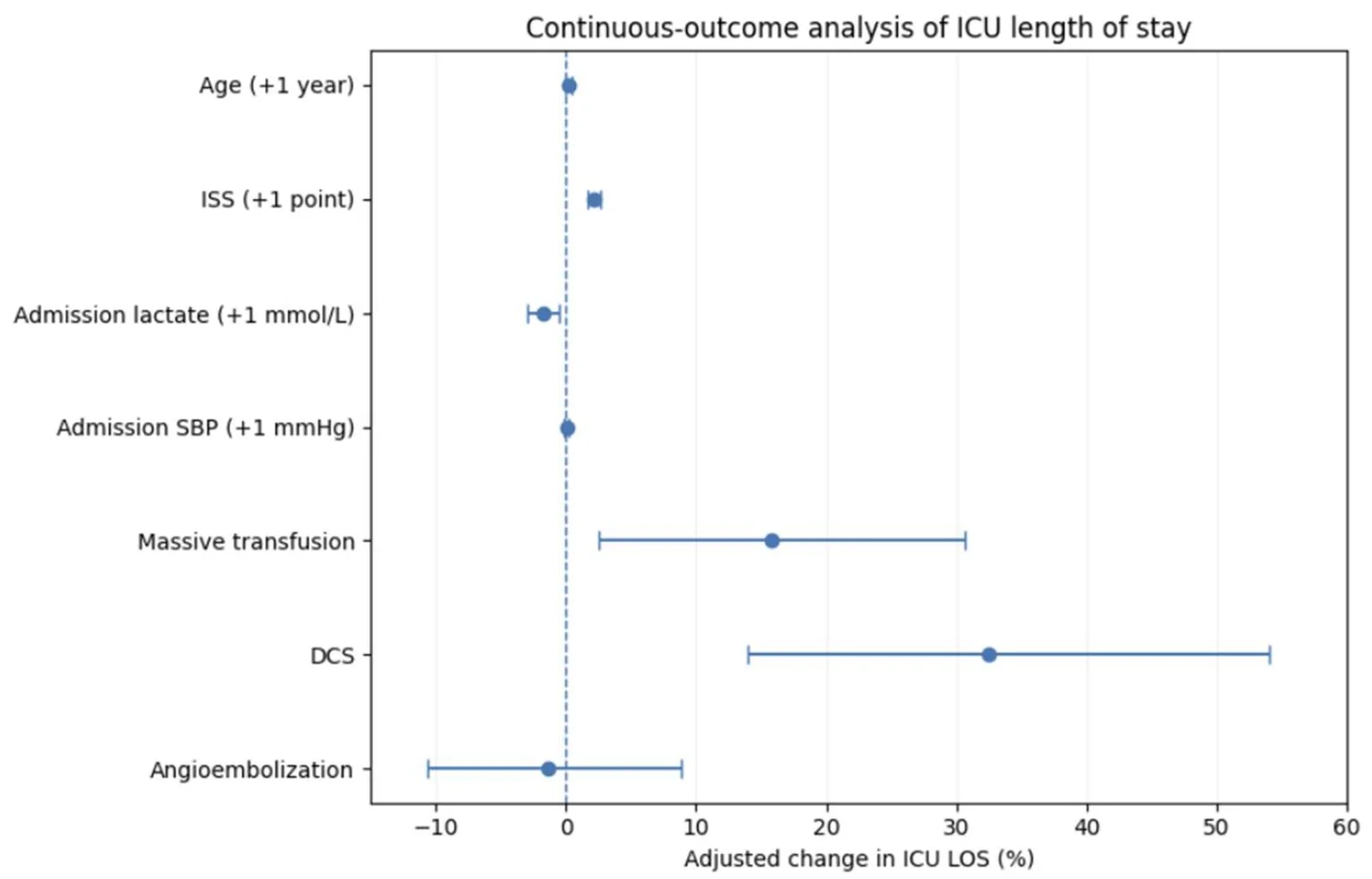
Sensitivity analysis using log-transformed ICU length of stay as a continuous outcome. Horizontal lines indicate 95% confidence intervals. Points represent adjusted percentage changes in ICU length of stay derived from multivariable linear regression of log-transformed ICU LOS. The vertical reference line indicates no change (0%). Estimates were mutually adjusted for age, ISS, admission lactate, admission SBP, massive transfusion, DCS, and angioembolization. Abbreviations: DCS, damage-control surgery; ICU, intensive care unit; ISS, Injury Severity Score; LOS, length of stay; SBP, systolic blood pressure.

**Figure 4:**
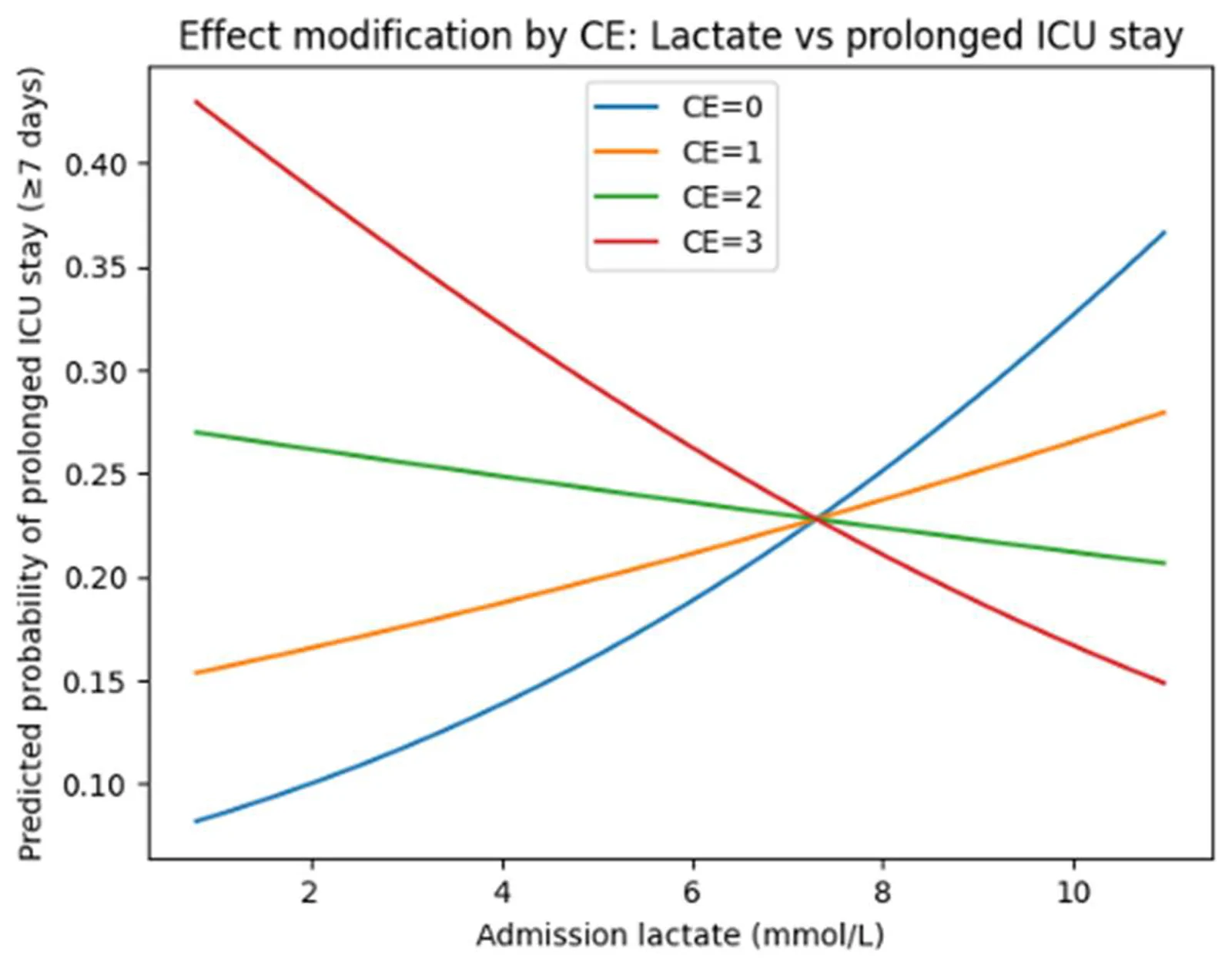
Prognostic heterogeneity in the association between admission lactate and prolonged ICU hospitalization across composite laboratory-based vulnerability score (CE) categories. Predicted probabilities of prolonged ICU stay (≥7 days) are shown across admission lactate levels for CE scores of 0–3, as estimated from the multivariable logistic regression model containing the CE-by-lactate interaction. Abbreviations: CE, composite admission laboratory-based vulnerability score; ICU, intensive care unit.

**Figure 5:**
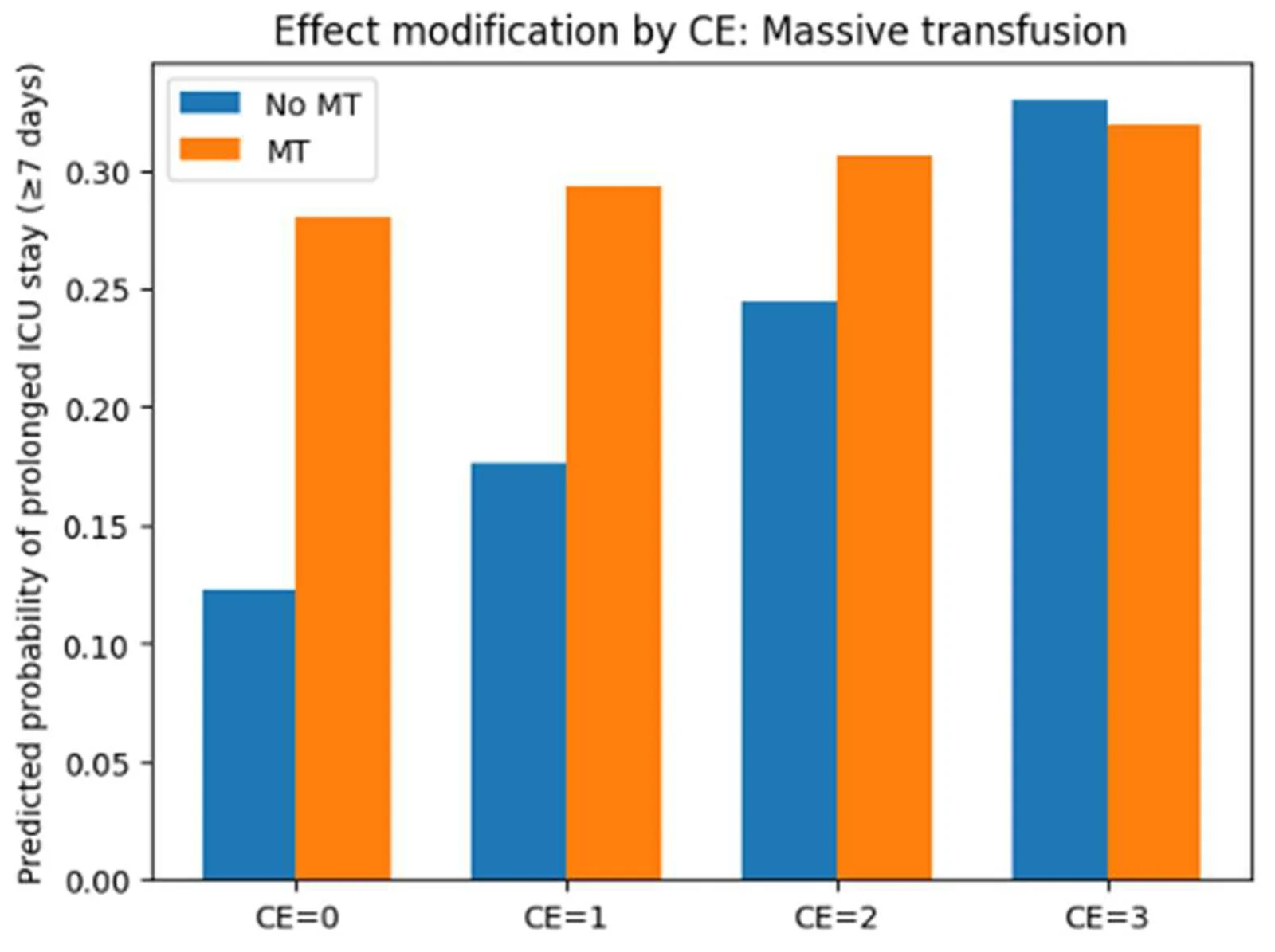
Prognostic heterogeneity in the association between massive transfusion and prolonged ICU hospitalization across composite laboratory-based vulnerability score (CE) categories. Predicted probabilities of prolonged ICU stay (≥7 days) are shown by massive transfusion status across CE scores of 0–3, as estimated from the multivariable logistic regression model containing the CE-by-massive-transfusion interaction. MT denotes receipt of massive transfusion, and No MT denotes no massive transfusion. Abbreviations: CE, composite admission laboratory-based vulnerability score; ICU, intensive care unit; MT, massive transfusion.

**Table 1:** Baseline characteristics by prolonged ICU stay (≥7 days).

Variable	ICU < 7 Days (*n* = 1881)	ICU ≥ 7 Days (n = 757)	*p* Value
Age, years	50.89 ± 20.12	54.24 ± 19.14	<0.001
ISS, points	17.21 ± 9.53	24.35 ± 9.88	<0.001
Admission lactate, mmol/L	3.48 ± 3.38	4.73 ± 3.62	<0.001
Admission SBP, mmHg	102.21 ± 26.18	92.51 ± 28.23	<0.001
Massive transfusion, *n* (%)	162 (8.6)	192 (25.4)	<0.001
Damage control surgery, *n* (%)	87 (4.6)	139 (18.4)	<0.001
Angioembolization, *n* (%)	386 (20.5)	203 (26.8)	<0.001

Data are presented as mean ± standard deviation or *n* (%), as appropriate. Abbreviations: ICU, intensive care unit; ISS, Injury Severity Score; SBP, systolic blood pressure.

**Table 2:** Multivariable associations with prolonged ICU stay (≥7 days).

Variable	Adjusted OR	95% CI	*p* Value
Age (years)	1.01	1.01–1.02	<0.001
ISS	1.06	1.05–1.08	<0.001
Lactate	1.01	0.98–1.04	0.495
SBP	1.00	0.99–1.00	0.051
Massive transfusion	1.52	1.09–2.12	0.013
Damage control surgery	2.35	1.60–3.46	<0.001
Embolization	0.97	0.76–1.24	0.797

The model included 2146 complete-case patients, of whom 611 (28.5%) had ICU LOS ≥ 7 days. Abbreviations: CI, confidence interval; ISS, Injury Severity Score; OR, odds ratio; SBP, systolic blood pressure.

**Table 3:** Sensitivity analysis using prolonged ICU stay ≥10 days.

Variable	Adjusted OR	95% CI	*p* Value
Age (years)	1.01	1.01–1.02	<0.001
ISS	1.06	1.05–1.08	<0.001
Lactate	1.02	0.99–1.05	0.224
SBP	1.00	0.99–1.00	0.174
Massive transfusion	1.56	1.10–2.22	0.012
Damage control surgery	1.88	1.27–2.79	0.002
Angioembolization	0.85	0.65–1.12	0.239

The model included 2146 complete-case patients, of whom 427 (19.9%) had ICU LOS ≥ 10 days. Abbreviations: CI, confidence interval; ISS, Injury Severity Score; OR, odds ratio; SBP, systolic blood pressure.

**Table 4:** Likelihood ratio tests and interaction term estimates.

Interaction Term	*N*	LR χ^2^	df	LR *p*	Interaction OR	95% CI	*p* (Term)
CE × massive transfusion	2146	4.35	1	0.037	0.70	0.50–0.98	0.036
CE × lactate	2146	44.13	1	<0.001	0.90	0.87–0.93	<0.001
CE × SBP	2146	7.61	1	0.006	1.01	1.00–1.01	0.006

Note: Lactate and SBP interactions use centered variables; interaction ORs reflect per-unit change in lactate and per mmHg change in SBP, respectively. Abbreviations: CE, composite laboratory-based vulnerability score; CI, confidence interval; LR, likelihood ratio; OR, odds ratio; SBP, systolic blood pressure.

**Table 5:** CE score–specific associations with prolonged ICU stay (≥7 days).

CE Score	Lactate +1 mmol/L, OR (95% CI)	*p* Value	SBP −10 mmHg, OR (95% CI)	*p* Value	Massive Transfusion, OR (95% CI)	*p* Value
0	1.12 (0.99–1.26)	0.065	1.03 (0.90–1.17)	0.659	2.71 (1.12–6.52)	0.027
1	1.06 (0.99–1.15)	0.127	0.99 (0.91–1.08)	0.818	2.00 (1.16–3.45)	0.013
2	1.01 (0.96–1.07)	0.719	0.96 (0.88–1.04)	0.338	1.48 (0.93–2.37)	0.100
3	0.96 (0.90–1.03)	0.236	0.93 (0.82–1.05)	0.250	1.10 (0.52–2.30)	0.802

Abbreviations: CE, composite admission laboratory score; CI, confidence interval; OR, odds ratio; SBP, systolic blood pressure.

## Data Availability

The data that support the findings of this study are available from the corresponding author upon reasonable request, subject to institutional and ethical restrictions.

## Supplementary Materials

The following supporting information can be downloaded at https://www.mdpi.com/article/10.3390/medicina62101832/s1, Figure S1: Patient selection for the primary analytic cohort; Table S1 Panel A: Availability of covariate data in the primary cohort (*n* = 2638); Table S1 Panel B: Availability of covariate data in the additional mortality sensitivity cohort (*n* = 881); Table S2: Adjusted associations with prolonged ICU stay (≥7 days) among hospital survivors (*n* = 688).
