# Supplementary material for: Evaluation of Prognostic Factors for Prolonged Intensive Care Unit Hospitalization After Trauma: Roles of Hemorrhagic Burden and Admission Laboratory Abnormalities
Source: Medicina (Kaunas). 2026 Sep 23;62(10):1832. doi: 10.3390/medicina62101832 (PMC13707285; doi:10.3390/medicina62101832)
Supplement: Supplementary file 1 [file medicina-62-01832-s001.zip › medicina-4550908-supplementary.pdf]

Supplementary Table S1-Panel A. Availability of covariate data in the primary cohort (n=2,638).

Variable	Available for analysis, n (%)	Unavailable for analysis, n (%)
Age	2,638 (100.0)	0 (0.0)
ISS	2,515 (95.3)	123 (4.7)
Admission lactate	2,214 (83.9)	424 (16.1)
Admission SBP	2,637 (100.0)	1 (<0.1)
Massive transfusion	2,638 (100.0)	0 (0.0)
DCS	2,638 (100.0)	0 (0.0)
Angioembolization	2,638 (100.0)	0 (0.0)
Complete information for all seven covariates	2,146 (81.3)	492 (18.7)

Notes: Percentages were calculated using the 2,638 patients in the primary cohort as the denominator. Missingness overlapped across variables; therefore, variable-specific counts should not be summed to obtain the number excluded from the complete-case model. One initially nonnumeric lactate entry was resolved by verification and included as an available measurement. Lactate values reported with inequality signs were entered at the reported numerical limits. Blank 24-hour packed red blood cell quantities were coded as no massive transfusion; availability for this derived variable therefore reflects this coding rule.

Abbreviations: DCS, damage-control surgery; ISS, Injury Severity Score; SBP, systolic blood pressure.

Supplementary Table S1-Panel B. Availability of covariate data in the additional mortality sensitivity cohort (n=881).

Variable	Available, n (%)	Unavailable for analysis, n (%)
Age	881 (100.0)	0 (0.0)
ISS	870 (98.8)	11 (1.2)
Admission lactate	784 (89.0)	97 (11.0)
Admission SBP	881 (100.0)	0 (0.0)
Massive transfusion	872 (99.0)	9 (1.0)
DCS	881 (100.0)	0 (0.0)
Angioembolization	881 (100.0)	0 (0.0)
CE score	881 (100.0)	0 (0.0)
Complete information for all required covariates	769 (87.3)	112 (12.7)

Notes: Percentages were calculated using the 881 patients in the additional mortality sensitivity cohort as the denominator. Of these, 769 had complete data for all required covariates; after excluding 81 in-hospital deaths from the complete-case sample, 688 hospital survivors remained for the survivor-restricted analysis. Lactate was missing in 95 records; two additional records contained only an above-limit value and were excluded from analyses requiring an exact continuous value. Missingness overlapped across variables, so variable-specific counts should not be summed to obtain the number of excluded records.

CE availability indicates that all three component abnormalities could be classified, including laboratory values reported below a limit that permitted unambiguous classification.

Abbreviations: CE, composite admission laboratory-based vulnerability score; DCS, damage-control surgery; ISS, Injury Severity Score; SBP, systolic blood pressure.

Supplement Table S2. Adjusted associations with prolonged ICU stay (≥7 days) among hospital survivors (n=688).

variables	Adjusted OR (95% CI)	P value
Age, +1 year	1.022 (1.009–1.035)	<0.001
ISS, +1 point	1.083 (1.058–1.109)	<0.001
Admission lactate, +1 mmol/L	1.077 (1.009–1.150)	0.026
Admission SBP, +1 mmHg	1.002 (0.993–1.010)	0.689
Massive transfusion	2.42 (1.45–4.03)	<0.001
DCS	5.58 (3.07–10.17)	<0.001
Angioembolization	3.86 (1.86–8.01)	<0.001

Abbreviations: CI, confidence interval; DCS, damage-control surgery; ICU, intensive care unit; ISS, Injury Severity Score; OR, odds ratio; SBP, systolic blood pressure.

Note: All listed variables were entered simultaneously into the multivariable logistic regression model.

Supplementary Figure S1. Patient selection for the primary analytic cohort

Trauma registry records screened
January 1, 2006–December 31, 2021
n = 4,106

↓

Excluded
ICU length-of-stay data unavailable: n = 1,468

↓

Primary analytic cohort
n = 2,638

↓

ICU LOS <7 days	ICU LOS ≥7 days
n = 1,881	n = 757 (28.7%)

Abbreviation: ICU, intensive care unit; LOS, length of stay.

Note: This figure depicts the primary cohort selection directly supported by the analysis dataset. No age-based or early-mortality exclusion is shown because these exclusions are not reflected in the 2,638-patient primary cohort.
